# The fetal thymus has a unique genomic copy number profile resulting from physiological T cell receptor gene rearrangement

**DOI:** 10.1038/srep23500

**Published:** 2016-03-24

**Authors:** Anders Valind, C. Haikal, M. E. K. Klasson, M. C. Johansson, J. Gullander, M. Soller, B. Baldetorp, David Gisselsson

**Affiliations:** 1 Department of Clinical Genetics, Lund University BMC C13, Lund University, SE 221 84, Lund, Sweden; 2 Department of Oncology, Lund University University Hospital, SE 221 85, Lund, Sweden; 3 Department of Clinical Genetics, Skåne Regional and University Laboratories University Hospital, SE 221 85, Lund, Sweden; 4 Department of Pathology, Skåne Regional and University Laboratories University Hospital, SE 221 85, Lund, Sweden

## Abstract

Somatic mosaicism, the presence of genetically distinct cells within an organism, has been increasingly associated with human morbidity, ranging from being a cause of rare syndromes to a risk factor for common disorders such as malignancy and cardiovascular disease. Previous studies interrogating the normal prevalence of somatic mosaicism have focused on adults. We here present an estimate of the baseline frequency of somatic mosaic copy number variation (CNV) at the time around birth, by sampling eight different organs from a total of five fetuses and newborns. Overall we find a significantly lower frequency of organ specific (i.e. mosaic) CNVs as compared to adults (p = 0.003; Mann-Whitney U-test). The rate of somatic CNV in adults has been estimated to around 2.2 CNV per organ assayed. In contrast, after stringent filtering, we found no organ-private CNVs in fetuses or newborns with exception of the thymus. This organ exhibited a specific genome profile in the form of deletions resulting from polyclonal T-cell receptor rearrangements. This implies that somatic non-immune related CNVs, if present at birth, are typically confined to very small cell populations within organs.

Somatic mosaicism is increasingly acknowledged as an important factor behind human disease^1^,^2^,^3^,^4^. This is exemplified by recent publications connecting somatic copy number variants to an increased risk of cancer^3^,^5^ and to shorter overall survival^6^. To date, almost all published studies of somatic copy number variants (CNV) have focused on adults. It is therefore not known whether somatic variation at the copy number level is present at detectable levels already in utero, whether it is generated de novo after birth, or present in utero as low frequency clones that expand during the life of an individual. Another fundamental question is whether the baseline level of somatic CNV at birth differs between different parts of the human body. For example, it has been suggested that the human liver exhibits a high degree of somatic CNV in the form of aneuploidy even in small children^7^, but these data have not been independently validated. As there is a strong connection between age and the presence of somatic copy number variants in peripheral blood^8^, it is important to investigate whether this also holds for solid organs.

To address these issues, we set out to derive a first estimate of the level of somatic copy number mosaicism present around birth. To detect organ-private CNVs indicative of somatic variation^9^,^10^, we analyzed tissue obtained at perinatal autopsy from eight different organs from four fetuses and one newborn using high resolution whole genome genotyping arrays (Table 1). We also mapped the levels of intra-organ aneuploidy using well-established *in situ* methods.

## Results

The study was approved by the regional ethics review board as a specific project within a larger study on the role of CNVs in perinatal disease. Anonymized tissue samples were obtained at clinical autopsies of fetal and perinatal deaths, where cases were selected for studies of baseline CNV on the criteria that they should neither exhibit developmental anatomic abnormalities, nor maceration threatening DNA quality. After strict filtering with respect to DNA quality and genotyping performance (See Methods for details) 3–8 tissue types (heart, lung, liver, spleen, thymus, kidney, adrenal gland, and muscle) per patient remained for analysis. In order to minimize the risk of false positives, we used a stringent calling method. In short, we ran each sample on two different high resolution genomic arrays (Affymetrix Cytoscan HD with a total of 2.67 million makers and Illumina Human Omni Quad 5 M with a total of 4.3 million markers) and retained only aberrations that spanned at least 50 consecutive probes and required a 50% reciprocal overlap between the segments from the two arrays to call a CNV. The latter criterion has previously been adopted by the 1000 Genomes project^11^ and also used as a concordance measure for array-platform comparison^12^. Using these calling criteria followed by manual visual inspection of the raw data, we detected a total of only eight true organ-private CNVs, all deletions, present in three of the patients. These eight variants were all found in the thymus and were situated at T cell receptor (TCR) loci, thus representing physiological RAG-mediated somatic rearrangements during T cell ontogeny. As a second step, we then re-analyzed the data by lowering the threshold for inclusion to ten consecutive probes. This resulted in more putative variants for cross validation and manual visual inspection (23 putative variants for visual inspection using 50 probes as a threshold compared 85 using 10 probes). However, after these validation steps only one additional CNV was retained. This consisted of a small 5 kb deletion located in the *TCRD* locus in one of the individuals (case 4).

**Figure 1:**
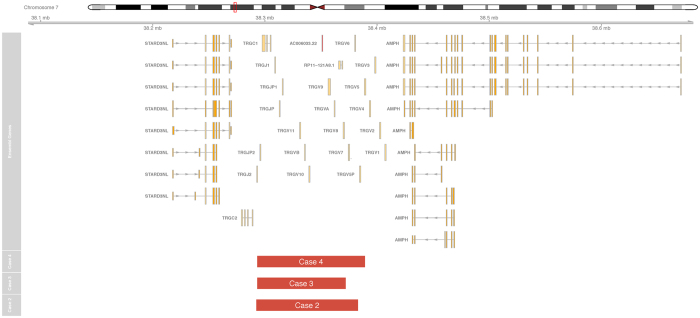
Recurrent *TCRG* deletions. RAG-mediated deletions at the T cell receptor gamma locus in 7p detected in three out of five individuals. The red bars denote the extension of the deletions in each of the cases using the stringent 50 probe threshold and the gene annotation track shows the location of *TCRG* genes.

**Figure 2:**
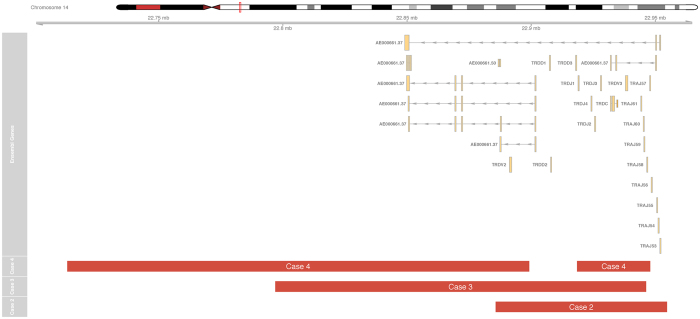
Recurrent *TCRD* deletions. RAG-mediated deletions at the T cell receptor delta locus in 14q detected in three out of five individuals using the stringent 50 probe threshold. Annotations are as in Fig. 1.

The three CNV-positive thymus samples all carried deletions in the *TCRG* and *TCRD* loci (Figs 1 and 2). One sample also had a deletion at the *TCRB* locus. The deletions in the *TCRD* loci were similar in breakpoints to rearrangements found in lymphoid neoplasms^13^. However, all thymus samples were polyclonal at TCR clonality analysis, inferring that the detected deletions resulted from a variety of different somatic rearrangements leading to loss of overlapping genomic segments. A validation set of five additional thymus samples from a second set of fetuses from the third trimester all exhibited similar deletions in *TCRG* and *TCRD* (Table S1). Our findings of a preferential involvement of the *TCRG* and *TCRD* genes are in line with earlier work showing that most T cells have genomically reordered gamma/delta receptors even if they have an alpha/beta-phenotype^14^. It is also consistent with the sequential ordering of rearrangements of the T cell receptor loci, where *TCRD* and *TCRG* rearrange before *TCRB* and *TCRA*^15^. Even though our findings of thymus-specific TCR deletions are not entirely surprising considering that TCR rearrangements are well known to occur in utero, our data for the first time show a human somatic tissue type to have a recurrent unique copy number profile.

**Figure 3:**
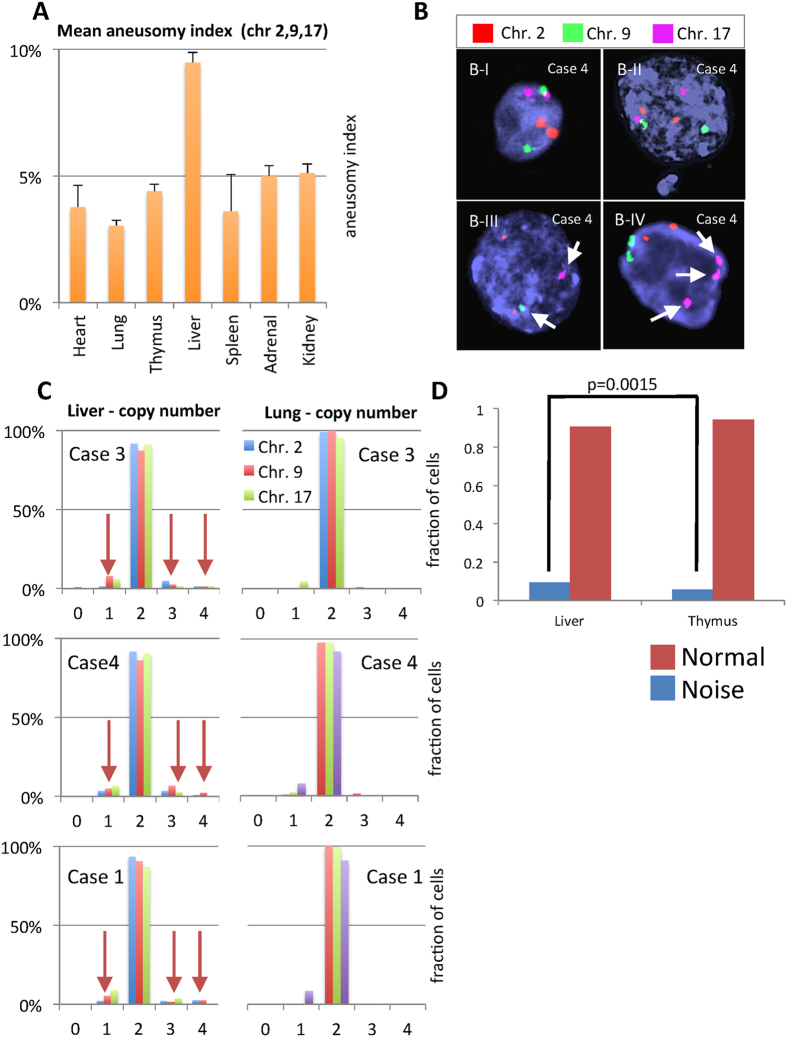
Intraorgan variation mapping using FISH. (**A**) Mean aneusomy index (AI) for all tissue types tested in cases 1, 3 and 4. For each tissue sample and chromosome, >200 nuclei were scored. The graph clearly shows a higher level of aneusomy in the liver. (**B**) Examples of FISH signal configurations that were scored as normal (B-I and B-II) as well as monosomic (B-III) and trisomic (B-IV) using the single probe method. (**C**) A comparison of chromosome number distributions for the liver (highest mean AI) and lung (lowest mean AI) for each of these cases, using single probe FISH. (**D**) The dual probe-based estimation of noise levels in the liver and the thymus for two samples (Case 1 and 4), showing that there is a significant increase (p-value from Fisher’s exact test) of false positive signals in the liver compared to the thymus samples. No true positive (aneusomic cells were detected in either tissue) and the number of true negative (disomic cells) were similar.

In addition to studying inter-organ variation, we also mapped intra-organ variation at the whole chromosome level in the three cases where cell nuclei were best preserved post mortem (cases 1, 3 and 4). The liver has been previously reported to harbor physiological chromosome copy number variation even in young individuals^7^. Using standard single color FISH we indeed found an elevated rate of aneuploidy in hepatocytes compared to cells in other organs (Fig. 3A–C). However, using a stringent, dual color FISH-method, previously shown to eliminate false positive noise signals^16^, liver cells failed to show an elevated prevalence of chromosome copy number changes. Instead false positive noise levels were considerably higher in the liver than in thymus samples used as a comparison (Fig. 3D). These findings are in concordance with a recent single cell sequencing study failing to show a higher frequency of aneuploidy in liver cells compared to keratinocytes^17^. In line with its role in post-natal hepatocyte differentiation and in contrast to adult livers^18^, there was no evidence of polyploidy in our fetal/neonatal liver samples, using either FISH or quantitative flow cytometry (DNA-FCM) (Fig. S1). We conclude that liver cells are no more aneuploid than most other somatic cells in fetuses and newborns. For the other organs assayed, the level of whole chromosome aneuploidy was at a level comparable with the noise inherent in single probe FISH (Fig. 3C).

## Discussion

One important limitation of our study is that we were unable to obtain tissue samples from all major organ systems. In particular, there was a lack of samples from the central nervous system (CNS) as perinatal autopsy guidelines stipulate that the entire brain should be subjected to neuropathological analysis. Including tissue from the CNS was therefore not considered ethical within the frames of the present study protocol, as it could interfere with the diagnostic process. The lack of data from the CNS is particularly regrettable as several studies have shown a wide variety of somatic variation confined to the brain^19^,^20^,^21^,^22^,^23^. Another weakness of the present study is its small sample size; this is both due to the difficulty of obtaining post mortem fetal tissue samples of sufficient quality for genome-wide analysis as well as the cost of that analysis. Nevertheless, our study contains a similar number of patients as the three previous studies of interorgan variation post mortem in adults^9^,^10^,^24^. Finally it must be noticed that our study can not differentiate between a complete absence of somatic variation within an organ and its presence in a small fraction (<10%) of cells within that organ.

With these limitations in mind, our data still reinforce the notion that detectable somatic mosaicism at the copy number level is an age-related phenomenon and establishes that this applies also outside the hematopoietic system. We also conclude that earlier reports of aneuploidy in the human liver seem to be biased by method specific noise and that liver cell polyploidy appears to be a post-natal phenomenon. This is in line with earlier reports focusing on somatic copy number variation within a single tissue type, i.e. peripheral blood, showing that the presence of mosaic events increase linearly with age^3^,^5^,^25^, although these studies only included samples from adult patients. There are very few studies focusing on somatic mosaicism within solid organs, possible due to the paucity of material^9^,^10^,^24^. The only comparable study in terms of array resolution found 2.2 CNV/organ assayed^9^, a significantly higher frequency than we detected in tissues outside the thymus (p = 0.003; Mann-Whitney U-test). It is possible that our stringent cross-platform validation criteria led to failure to ascertain some CNVs due to their small size and/or low prevalence in tissue. However, the fact that we detected rearrangements in TCR loci down to 5 kb in the thymus, events that are subclonal within this organ, argues against this. As high resolution whole genome genotyping arrays detect subclonal variation present in as low as 10% of cells in the sample assayed^26^, larger subpopulations carrying unique CNVs would most probably have been detected if present.

In the context of somatic mosaicism, the best-studied organ system besides human blood is probably the skin, assumedly due to the ease of getting access to material. Here, recent work has shown that smaller copy number variants can be present at very low allele frequencies^27^ and that the frequency of aneuploidy in human keratinocytes is at most a few percent^17^. The skin is presumably exposed to exogenous mutagens more often than other organs, resulting in a higher mutation rate and this may effect clonal dynamics^28^, possibly causing a larger age-dependent accumulation effect of somatic mosaicism than in other organs. Interestingly, another known mutagen, cigarette smoke, has been associated with an increase in mosaic loss of chromosome Y in peripheral blood in men^29^. Regarding somatic copy number variation at low frequencies at birth, recent studies have shown that low grade parental mosaicism can sometimes be transmitted, i.e. present in both the soma and the germline^30^. This shows that–in situations associated with genetic disease – somatic copy number variants can form during the very first stages of embryogenesis, followed by age dependent clonal expansion, possibly modulated by various environmental cues.

To summarize, we showed that there was no detectable somatic copy number mosaicism beyond physiological immunological rearrangements, in a total of 28 fetal/neonatal organs that passed stringent DNA quality criteria. Our data provide the first experimental evidence that detectable somatic CNVs are typically postnatal phenomena in individuals without congenital genetic disease.

## Methods

### Organ Sampling and DNA extraction

Autopsy, biobanking and genetic analyses were performed per clinical routine after informed consent from next of kin. All experiments were performed according to the ethical guidelines for research at the Lund University Faculty of Medicine. The study protocol was approved by the Regional Ethics Committee (No. L2012/405). Per this protocol, 5 mm tissue samples from multiple organs were immediately frozen under a serial number, which allowed safe transmission of basic clinical data (Table 1) for comparison to genomic results while keeping patient identity confidential. No case was included for research purposes only. Patients with malformation syndromes of potential genetic origin as well as severely macerated foetuses were excluded from the study. DNA was extracted from frozen samples using the DNeasy Blood and Tissue Kit (QIAGEN, Hilden, Germany).

### Quality Control on the Discovery Platform

The Affymetrix Cytoscan HD uses three different metrics to evaluate the quality of the generated data: SNPQC, the median absolute value pairwise difference (MAPD), and Waviness-SD. SNPQC is a metric measuring the separation of the genotype cluster that gives information on the quality of the genotyping for the subset of probes on the Cytoscan Array that are genotypeable. MAPD measures local variation in the log2-ratio, while the Waviness-SD measures variation in log2-ratio over larger genomic space, as described in the user manual for the Affymetrix Chromosome Analysis Suite sofware^31^. As the Waviness-SD metric is sensitive to the presence of true copy number variants we used only the MAPD and SNPQC while evaluating the quality of the generated data. All samples that were below the thresholds for MAPD and SNPQC as set by Affymetrix where excluded from downstream analysis. The MAPD and SNPQC values for all samples assayed are presented in Supplemental Table 2.

### Calling process for organ specific CNVs

The Affymetrix Cytoscan HD data and the Illumina 5 M Omni Quad data were loaded into two platform specific Nexus Copy Number 7.5 projects, and then normalized and filtered according to the default setting for the corresponding array type. They were subsequently segmented using the FASST2-SNP segmentation method using the standard parameter for calling thresholds. FASTT2-SNP is a Hidden Markov Model-based segmentation algorithm that incorporates both the Log2-ratio information as well as the alternate allele frequency when calling copy number variants. All segments where then exported and, using custom python scripts, segments that spanned less than 50 (first analysis) and 10 (second analysis) consecutive markers were removed together with non-copy number variants (i.e. allelic imbalances and LOH-events). The filtered files were then converted to .bed files for downstream processing with the Bedtools-suite^32^, using the following steps:

1. Merging of directly adjacent segments of the same type.
2. Per-patient pairwise within-organ, between platform-intersection, with a minimum criterion of 50% reciprocal overlap.
3. Per-patient all-organ intersection (using bedtools multiinter) to remove variants present in all organs (i.e. germline variants)

This was followed by visual inspection of the remaining segments and the raw probe data in Nexus Copy Number 7.5.

### Detection of aneuploidy

DNA-FCM analysis was performed by routine methods, as described^33^, on formalin-fixed paraffin embedded tissue with >90% intact nuclei by microscopic inspection. Single and dual color interphase FISH was performed as described previously^16^,^34^. In short, touch preparations were made by gently pressing a small frozen tissue sample onto a microscopic slide using a surgical forceps. This slide was subsequently washed and pepsinated and treated with 1% formamide at room temperature. After this the slides were dehydrated using a 75–80–100% ethanol series and air-dried followed by application of the probe mixture and hybridization over night. We defined true aneusomy as a non-disomic signal pattern consisting of the same number of signals for both probes located to the same chromosome. Patterns consisting of an uneven number of dual color signals were defined as noise and would have represented false positives using single color FISH.

### TCR clonality analysis

This was performed as described in^35^, using four validated multiplexed single tube PCR on DNA from the same extract as was genotyped on the array platforms. The First PCR reaction targeted the *TCRG* locus (as well as IGH, t(14;18) and t(11;14)). The second multiplex reaction targeted *TCRD*-rearrangements and the last two targeted the *TCRB* locus. For detailed information about primers used we refer to Dictor *et al*.^35^.

## Additional Information

**How to cite this article**: Valind, A. *et al*. The fetal thymus has a unique genomic copy number profile resulting from physiological T cell receptor gene rearrangement. *Sci. Rep.* **6**, 23500; doi: 10.1038/srep23500 (2016).

## Supplementary Material

Supplementary Information

## Figures and Tables

**Table 1:**
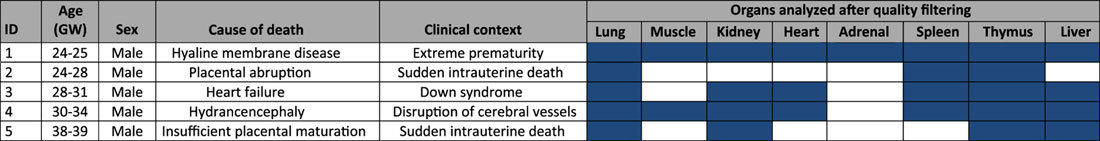
Patient characteristics based on autopsy findings with age given in gestational weeks (GW), together with organs assayed for each patient.

Blue cells in the table indicate organs that passed all quality controls and were analyzed by genomic array.
