# Supplementary material for: The fetal thymus has a unique genomic copy number profile resulting from physiological T cell receptor gene rearrangement
Source: Sci Rep. 2016 Mar 24;6:23500. doi: 10.1038/srep23500 (PMC4806331; doi:10.1038/srep23500)
Supplement: Supplementary Information [file srep23500-s1.pdf]

Supplemental Information for

The fetal thymus has a unique genomic copy number profile resulting from physiological T cell receptor gene rearrangement

Anders Valind[1*], C. Haikal[1], M.E.K. Klasson[1], M. C. Johansson[2], J. Gullander[1], M. Soller[3], B. Baldetorp [2], David Gisselsson[1,4]

Supplemental Figure 1: Results from DNA-FCM for one of the thymus samples (ST1) from the second set analyzed, showing only a diploid G1 peak.

Supplemental Table 1: Deletions present at the *TCRG* and *TCRD* loci in the second set of thymus samples assayed (n=5) on the Cytoscan HD.

Supplemental Table 2: Quality control values for all samples included in the analysis.

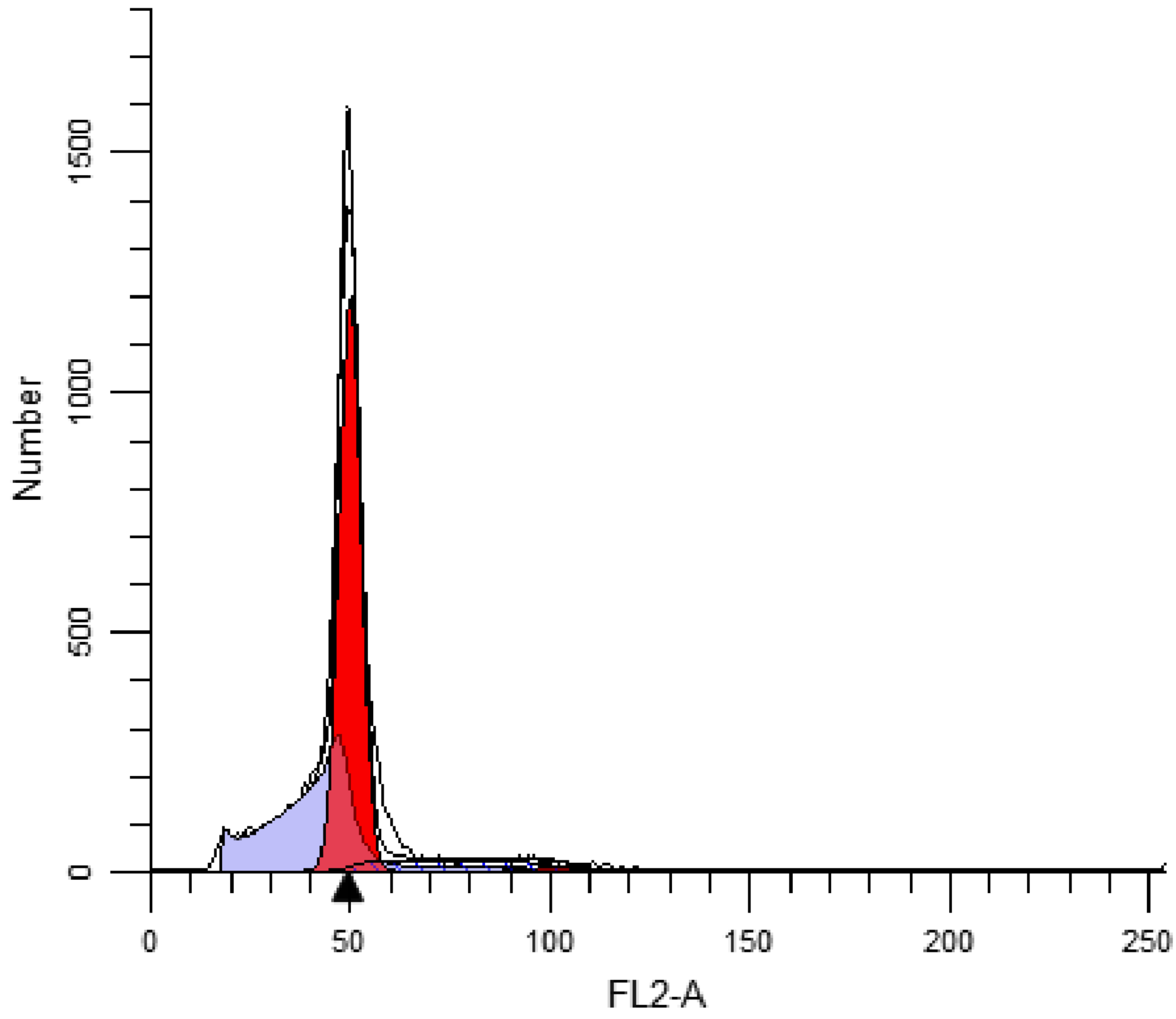

<u>Figure S2:</u> Quantitative flow cytometry for sample ST1 showing only a diploid G1 peak

TCRG

case_id	chromosome	start	stop	type
ST1	7	38294982	38400944	Loss
ST2	7	38261216	38395208	Loss
ST3	7	38209524	38476836	Loss
ST4	7	38292662	38394857	Loss
ST5	7	38292593	38394857	Loss

TCRD

case_id	chromosome	start	stop	type
ST3	14	22313320	22703926	Loss
ST2	14	22331351	22358285	Loss
ST2	14	22379131	22410495	Loss
ST1	14	22563613	22598376	Loss
ST2	14	22563613	22704030	Loss
ST5	14	22563613	22969566	Loss
ST3	14	22616042	22944507	Loss
ST1	14	22624362	22913130	Loss
ST3	14	22722723	22795091	Loss
ST2	14	22730240	23036343	Loss
ST3	14	22837373	23070224	Loss
ST1	14	22917632	22959362	Loss

Case	Organ	SNPQC (>= 15)	MAPD (<= 0.25)
1	Lung	26,8	0,16
	Muscle	23,6	0,21
	Kidney	24,8	0,18
	Heart	23,5	0,18
	Adrenal	24	0,18
	Spleen	26	0,18
	Thymus	23,7	0,17
	Liver	24	0,2
2	Lung	18	0,22
	Spleen	15	0,24
	Thymus	16	0,25
3	Spleen	17,2	0,22
	Kidney	18,2	0,2
	Heart	22	0,18
	Thymus	22	0,18
	Liver	17	0,2
	Lung	21	0,17
4	Lung	27	0,16
	Muscle	26	0,16
	Thymus	25	0,17
	Spleen	24	0,17
	Kidney	19	0,18
	Liver	24	0,16
	Heart	23	0,17
5	Lung	21,8	0,19
	Kidney	17,3	0,21
	Thymus	15,7	0,24
	Spleen	18,9	0,2
